# Analysis of antifungal resistance genes in *Candida albicans* and *Candida glabrata* using next generation sequencing

**DOI:** 10.1371/journal.pone.0210397

**Published:** 2019-01-10

**Authors:** Kathrin Spettel, Wolfgang Barousch, Athanasios Makristathis, Iris Zeller, Marion Nehr, Brigitte Selitsch, Michaela Lackner, Peter-Michael Rath, Joerg Steinmann, Birgit Willinger

**Affiliations:** 1 Department of Laboratory Medicine, Division of Clinical Microbiology, Medical University of Vienna, Vienna, Austria; 2 Division of Hygiene and Medical Microbiology, Medical University of Innsbruck, Innsbruck, Austria; 3 Institute of Medical Microbiology, University Hospital Essen, University of Duisburg-Essen, Essen, Germany; 4 Institute of Clinical Hygiene, Medical Microbiology and Infectiology, Paracelsus Medical University, Nuremberg, Germany; Louisiana State University, UNITED STATES

## Abstract

**Introduction/Objectives:**

An increase in antifungal resistant *Candida* strains has been reported in recent years. The aim of this study was to detect mutations in resistance genes of azole-resistant, echinocandin-resistant or multi-resistant strains using next generation sequencing technology, which allows the analysis of multiple resistance mechanisms in a high throughput setting.

**Methods:**

Forty clinical *Candida* isolates (16 *C*. *albicans* and 24 *C*. *glabrata* strains) with MICs for azoles and echinocandins above the clinical EUCAST breakpoint were examined. The genes *ERG11*, *ERG3*, *TAC1* and *GSC1* (*FKS1*) in *C*. *albicans*, as well as *ERG11*, *CgPDR1*, *FKS1* and *FKS2* in *C*. *glabrata* were sequenced.

**Results:**

Fifty-four different missense mutations were identified, 13 of which have not been reported before. All nine echinocandin-resistant *Candida* isolates showed mutations in the hot spot (HS) regions of *FKS1*, *FKS2* or *GSC1*. In *ERG3* two homozygous premature stop codons were identified in two highly azole-resistant and moderately echinocandin-resistant *C*. *albicans* strains. Seven point mutations in *ERG11* were determined in azole-resistant *C*. *albicans* whereas in azole-resistant *C*. *glabrata*, no *ERG11* mutations were detected. In 10 out of 13 azole-resistant *C*. *glabrata*, 12 different potential gain-of-function mutations in the transcription factor *CgPDR1* were verified, which are associated with an overexpression of the efflux pumps CDR1/2.

**Conclusion:**

This study showed that next generation sequencing allows the thorough investigation of a large number of isolates more cost efficient and faster than conventional Sanger sequencing. Targeting different resistance genes and a large sample size of highly resistant strains allows a better determination of the relevance of the different mutations, and to differentiate between causal mutations and polymorphisms.

## Introduction

*Candida* spp. has emerged as an important pathogen causing bloodstream infections associated with a high mortality. *C*. *albicans* and the less susceptible *C*. *glabrata* are the most common species causing candidemia and candidiasis [1, 2]. Echinocandins and azoles play an important role in the therapeutic management of invasive candidiasis. In recent years, *Candida* isolates with acquired resistance to azoles and echinocandins have been reported more frequently [3, 4]. Therefore, antifungal susceptibility testing and the detection of mutations in resistance genes are becoming increasingly important to detect antifungal resistance and determine the underlying resistance mechanisms.

Echinocandins inhibit the glycosyltransferase 1,3-β-D-glucan synthase (*FKS*) non-competitively. This enzyme is responsible for the biosynthesis of the oligosaccharide 1,3-β-D-glucan, an important structural component of the fungal cell wall [5]. Decreased susceptibility to echinocandins is associated with target mutations in the hot spot (HS) regions of Fks proteins, which represent the putative binding domain of the echinocandins. Point mutations in these regions can reduce the affinity of the echinocandins to 1,3-β-D-glucan synthase [3, 6, 7].

The pharmacological target of azoles is the enzyme 14-α-demethylase (encoded by *ERG11*), an important enzyme in ergosterol biosynthesis. Acquired resistance to azoles may be caused by several mechanisms. Mutations of the pharmacological target are able to change the enzyme’s structure and may result in reduced binding affinity of the azoles to Erg11p [8, 9]. Frequently, efflux pumps reduce the intracellular accumulation of azoles. The increased efflux is based on overexpression of *CDR1*/*CDR2* (Candida Drug Resistance) and *MDR1* (Multi Drug Resistance). Gain-of-function mutations in the transcription factors *TAC1* and *CgPDR1* can lead to higher gene expression of drug efflux pumps [10–12].

Loss-of-function mutations in the enzyme Erg3p are another mechanism of azole resistance. In addition to the inhibition of Erg11p, azoles cause a metabolic bypass resulting in the accumulation of toxic concentrations of 14α-methyl-3,6-diol. This metabolite blocks fungal growth. Loss-of-function mutations in *ERG3* inhibit the conversion of 14α-methylfecosterol to toxic 14α-methyl-3,6-diol thereby decreasing azole efficacy. Additionally, the precursor 14α-methylfecosterol can be used to substitute ergosterol [13, 14].

The purpose of this study was to investigate clinical isolates of *C*. *albicans* and *C*. *glabrata* showing either echinocandin or azole resistance or both *in vitro*, and to correlate the resistant phenotypes with described mutations in resistance genes.

Despite generating much data, whole genome sequencing has crucial disadvantages like low coverage levels and a high data analysis burden. Sanger Sequencing is not suitable either, because of the time-consuming process and high costs per sequenced base. Targeted resequencing design offers many advantages over conventional sequencing approaches for the parallel sequencing of a high number of isolates, like higher reliability, the fast sequencing process and more manageable data analysis. Next generation sequencing (NGS) is a very efficient tool to study a set of genes that are known to be involved in antifungal-resistance in a comprehensive strain set. Therefore, NGS based on targeted resequencing design was used to investigate the underlying resistance mechanisms in our clinical isolates.

We sequenced the genes involved in echinocandin resistance (*GSC1* in *C*. *albicans* and *FKS1* and *FKS2* in *C*. *glabrata)* and the genes involved in azole resistance (*ERG11*, *TAC1* and *ERG3* in *C*. *albicans* and *ERG11* and *CgPDR1* in *C*. *glabrata)*. The aim of this study was not only to detect established but also to identify novel point mutations associated with echinocandin or azole-resistance in the described resistance genes.

## Methods

### Sampling and antifungal susceptibility testing

Forty isolates obtained from specimens such as swabs (4), sterile fluids (8), blood cultures (12), a central venous catheter (1), as well as urine (3), feces (4), sputum (1) as well as not specified (7) from various centres in Austria and Germany were investigated. In addition, the susceptible strains ATCC 90030 and ATCC Y33.90 for *C*. *glabrata*, and ATCC 90028, ATCC 10231, as well as the azole-resistant ATCC 64124 for *C*. *albicans* were included as controls for the validation of the sequencing process. Antifungal susceptibility testing using the broth microdilution method was performed for all strains and *C*. *parapsilosis* ATCC 22019 and *C*. *krusei* ATCC 6258 as control strains as described by the European Committee of Antimicrobial Susceptibility Testing (EUCAST E.DEF 7.3 December 2015) [15]. Minimal inhibitory concentrations (MIC) were determined for anidulafungin, micafungin and caspofungin, as well as for fluconazole, posaconazole, voriconazole, itraconazole and isavuconazole. Strains with a MIC one to twofold dilutions above the clinical breakpoint for echinocandins were classified as borderline echinocandin resistant. Multi-resistant isolates were defined as resistant to all tested echinocandins and azoles.

### DNA extraction

Due to better quantitative and qualitative results in comparison to commercial DNA extraction kits, a modified SDS CTAB chlorophorm based method was used [16]. DNA extraction was performed from a 24h Candida culture on Sabouraud-dextrose agar (SAB). Mechanical lysis was carried out using 1mm silica spheres under addition of the detergents SDS (sodium dodecyl sulfate) and CTAB (cetyltrimethylammonium bromide) as well as proteinase K (Qiagen, Venlo, Netherlands). After adding chloroform-isoamylalcohol 24:1, the water-soluble polar layer was transferred to a new tube followed by precipitation with ammonium acetate and isopropanol and was subsequently washed using ethanol. The air-dried DNA was then resuspended in 10 mM Tris-EDTA buffer. After extraction, the amount of DNA was determined with Qubit 2.0 via the dsDNA HS kit (Life technologies, Carlsbad, California) and NanoDrop 2000c spectrophotometer (Thermo Scientific, Waltham, Massachusetts). Additionally, the ratios A260/280 and A260/230 were used to estimate the purity of the DNA.

### Next generation sequencing and library preparation

Sequencing was carried out using a targeted resequencing design on the MiSeq platform (Illumina, San Diego, California). Sequence analysis was performed for the whole gene sequence of *ERG11* and *ERG3*, the HS regions of *FKS1*, *FKS2* and *GSC1*, as well as relevant regions of *TAC1* and *CgPDR1* (Table 1). Sequencing of *ERG11* and *ERG3* was achieved using overlapping primers and subsequent assembly. The amplicon sequencing was based on the 16s protocol as described by Illumina [17]. PCR 1 was performed with locus-specific primers with the additional overhang sequence that is mandatory for sequencing with Illumina technology. Four of the 26 primer pairs published by Garnaud et al. were newly designed by Primer3 Tool because of the formation of hairpins and primer dimers (S1 and S2 Tables) [18, 19]. The library amplification was performed using the KAPA HiFi Hot Start Ready Mix Kit (Kapa Biosystems, Wilmington, Massachusetts), a high fidelity polymerase with proofreading activity which is well suited for the production of NGS-libraries [20]. 12.5 ng genomic DNA was added to the PCR-mix. Afterwards, the amplified PCR products of each isolate were pooled. The washing steps were based on Ampure Beads (Beckman Coulter, Brea, California). Subsequently, an index PCR was performed to tag the amplicons for identification of the different isolates after pooling. The DNA quantification of the PCR products was carried out using Qubit 2.0 via the dsDNA HS kit (Life technologies, Carlsbad, California). DNA was diluted to a concentration of 8pM for sequencing on the V2-Flowcell 2x250bp (Illumina, San Diego, California) and all isolates were pooled. The DNA library was denatured according to the protocol [17]. For quality control, the library was spiked with 5% PhiX DNA.

**Table 1:** Overview of the sequenced regions from *C*. *albicans* and *C*. *glabrata*.

Species	Gene	Gene length (bp)	Coordinates (bp)	Sequenced gene length (bp)
*C*. *albicans*	*GSC1*	5694	1752–2130; 3885–4273	768
	*TAC1*	2946	1879–2253; 2720- +166	768
	*ERG11*	1587	-71 - +22	1680
	*ERG3*	1161	-33 - +28	1222
		Total		4438
*C*. *glabrata*	*FKS1*	5592	1693–2075; 3831–4225	778
	*FKS2*	5694	1802–2198; 3935–4326	789
	*ERG11*	1602	-89 - +45	1736
	*CgPDR1*	3324	804–1168; 1526–1922; 2355–2753; 2968- +31	1549
		Total		4852

### Bioinformatic analysis

The quality of the NGS run was verified using the software FASTQC 0.11.4 [21]. The removal of low-quality bases was carried out with the Trimmomatic-0.35 software [22]. This tool also removed all reads under a minimum length of 90bp. In addition, the first 24bp were removed to exclude the primer sequences. The reads were assembled with Bowtie2-2.2.7 [23]. Subsequently, alignment to the reference sequence was carried out. The strains SC5314 for *C*. *albicans* and CBS138 for *C*. *glabrata* were used as reference sequences. The gene sequences were downloaded from www.candidagenome.org [24]. To determine variants from the reference sequence Samtools 0.1.19 and VarScan.v2.3.9 [25] were used. After this, SnpEff 4.270 was used to detect alterations causing amino acid substitutions. Finally, a visual validation of the mutations in the assembly files was performed to exclude bias variants. Table 2 shows the sources of the reference sequences. The sequences of the isolates have been deposited in the BioProject database under accession number PRJNA510782.

**Table 2:** Overview of the used reference sequences from Candida Genome Database (www.candidagenome.org).

Strain	Gene	Coordinates
*C*.*albicans* C5314 Assembly 22	GSC1 C1_02420C_A orf19.2929	Ca22chr1A_C_albicans_SC5314: 511662–505969
	TAC1 C5_01840C_A orf19.3188	Ca22chr5A_C_albicans_SC5314: 419345–416400
	ERG11 C5_00660C_A orf19.922	Ca22chr5A_C_albicans_SC5314: 149701–148115
	ERG3 C1_04770C_A orf19.767	Ca22chr1A_C_albicans_SC5314: 992782–991622
*C*.*glabrata* BS138	FKS1 CAGL0G01034g	ChrG_C_glabrata_CBS138: 93468–99059
	FKS2 CAGL0K04037g	ChrK_C_glabrata_CBS138 373375–379068
	PDR1 CAGL0A00451g	ChrA_C_glabrata_CBS138: 47557–50880
	ERG11 CAGL0E04334g	ChrE_C_glabrata_CBS138: 417189–415588

## Results

### EUCAST microdilution

Among the 19 *C*. *albicans* isolates, two were susceptible control strains, seven were resistant to azoles, six were echinocandin-resistant, two borderline echinocandin-resistant, and two were resistant against all tested echinocandins and azoles and were classified as multi-resistant isolates (Table 3). Among the 26 *C*. *glabrata* isolates, 13 were resistant to the tested azoles and three to the tested echinocandins. In addition, two isolates were borderline echinocandin-resistant, and six were multi-resistant. Each azole-resistant isolate exhibited a complete cross-resistance against all tested azoles with consistently high MIC values. In the case of echinocandins, with the exception of isolate Cg41, all strains showed a complete cross-resistance against echinocandins. Strain Cg41 revealed high MIC values for anidulafungin and caspofungin, but was susceptible to micafungin with a MIC value of 0.016 mg/l. Tables 3 and 4 show all MIC data.

**Table 3:** Potentially causal missense mutations and MIC values in *C*. *albicans* isolates.

ID	Resistance	Origin	Gene	Nucleotide Substitution	Aminoacid Substitution	Frequency (%)	Hot Spot	Literature	AFG	CAS	MFG	FLC	POS	ISA	ITC	VRC
Ca1	susceptible	ATCC 90028							0.008	0.016	0.016	0.25	0.032	<0.008	0.032	<0.016
Ca2	susceptible	ATCC 10231							0.008	0.016	<0.008	<0.125	0.016	<0.008	<0.008	<0.016
Ca3	azole-resistant	ATCC 64124	*TAC1*	2929A>G	N977D	49.26		Coste (2009) Coste (2006)	0.016	0.032	0.008	128	4	4	>16	8
			*ERG11*	214T>C	F72L	99.79		Favre (1999)								
			*ERG11*	394T>C	Y132H	99.9	HS 1	Favre (1999)								
			*ERG11*	1349G>A	G450E	99.73	HS 3	Favre (1999)								
			*ERG3*	503C>T	A353T	84.71		Morio (2012)								
			*ERG3*	986C>G	T329S	99.83		Morio (2012)								
Ca5	azole-resistant	not specified	*ERG11*	1309G>A	V437I	48.2	HS 3	Favre (1999)	0.016	0.032	0.016	>256	>32	>16	>16	>8
Ca6	azole-resistant	drainage fluid	*TAC1*	2810C>T	S937L	51.06			0.064	0.032	0.016	128	>32	>16	>16	>8
			*ERG11*	1309G>A	V437I	99.87	HS 3	Favre (1999)								
			*ERG3*	571T>C	S191P	99.0										
Ca8	azole-resistant	mouth swab	*TAC1*	2218A>G	N740D	100.0		Siikala (2010)	0.008	0.016	0.016	128	0.25	0.5	1	8
			*ERG11*	394T>C	Y132H	99.84	HS 1	Favre (1999)								
			*ERG11*	1349G>A	G450E	99.46	HS 3	Favre (1999)								
Ca9	azole-resistant	vaginal swab	*ERG11*	622G>A	E208K	50.0			0.064	0.064	0.032	>256	>32	>16	>16	>8
			*ERG11*	1574C>T	T525I	49.59										
			*ERG3*	782G>A	G261E	99.83										
Ca10	azole-resistant	mouth swab	*TAC1*	2939G>A	G980E	99.86		Coste (2009)	0.016	0.032	0.016	64	1	0.5	0.5	0.25
			*ERG11*	428A>G	K143R	99.84	HS 1	Manastir (2009) Flowers (2015)								
Ca11	azole-resistant	not specified	*TAC1*	1946G>A	G649D	99.85			0.016	0.032	0.016	64	0.5	0.25	1	0.5
			*TAC1*	2920T>C	F974L	99.7										
			*ERG11*	428A>G	K143R	99.86	HS 1	Manastir (2009) Flowers (2015)								
Ca13	echinocandin-resistant	bile drainage	*GSC1*	1922T>G	F641C	50.56	HS 1	Wiederhold (2011) Balashov (2006)*	0.125	0.125	0.064	0.25	0.032	<0.008	0.064	<0.016
			*GSC1*	1923C>T	F641C	49.17	HS 1	Wiederhold (2011) Balashov (2006)								
			*ERG3*	1057G>A	A353T	51.19		Morio (2012)								
Ca14	echinocandin-resistant	blood culture	*GSC1*	1922T>C	F641S	99.75	HS 1	Wiederhold (2011) Balashov (2006)*	0.5	1	1	0.25	0.032	<0.008	0.032	<0.016
			*ERG3*	1057G>A	A353T	51.74		Morio (2012)								
Ca15	echinocandin-resistant	not specified	*GSC1*	1933T>C	S645P	99.66	HS 1	Garnaud (2015)	0.5	2	1	0.25	0.032	<0.008	0.032	<0.016
Ca16	echinocandin-resistant	not specified	*GSC1*	1933T>C	S645P	48.64	HS 1	Garnaud (2015)	0.5	1	0.5	0.25	0.125	<0.008	0.064	<0.016
Ca17	echinocandin-resistant	jugularis catheter	*GSC1*	1933T>C	S645P	99.49	HS 1	Garnaud (2015)	0.5	2	2	0.25	0.032	<0.008	0.032	<0.016
Ca18	echinocandin-resistant	ascites	*GSC1*	1946C>A	P649H	99.85	HS1	Garcia-Effron (2008) Desnos-Ollivier (2008) Dudiuk (2015)	0.25	0.5	0.5	0.125	0.016	<0.008	0.016	<0.016
			*GSC1*	2086A>G	M696V	98.61	90 bp after HS1 30bp before HS3									
Ca19	borderline echinocandin-resistant	blood culture							0.064	0.064	0.064	0.25	0.125	<0.008	0.064	<0.016
Ca21	borderline echinocandin-resistant	blood culture							0.016	0.064	0.032	0.5	0.064	<0.008	0.125	<0.016
Ca12	multi-resistant	feces	*ERG3*	975C>A	Y325*	99.0			0.125	0.064	0.064	256	>32	>16	8	4
			*ERG3*	1057G>A	A353T	99.95		Morio (2012)								
Ca22	multi-resistant	feces	*ERG3*	570T>G	Y190*	99.84			0.25	0.5	0.125	>256	>32	>16	>16	>8
			*ERG3*	1057G>A	A353T	99.84		Morio (2012)								

* same position, different aminoacid substitution

**Table 4:** Potentially causal missense mutations and MIC values in *C*. *glabrata* isolates.

ID	Resistance	Origin	Gene	Nucleotide Substitution	Aminoacid Substitution	FREQ	Hot Spot	Literature	AFG	CAS	MFG	FLC	POS	ISA	ITC	VRC
Cg23	susceptible	ATCC 90030							0.064	0.032	0.016	16	1	0.5	0.5	0.25
Cg24	susceptible	ATCC Y33.90							0.032	0.032	0.016	4	0.25	0.125	0.125	0.25
Cg26	azole-resistant	blood culture	*PDR1*	3235G>A	G1079R	97.86		Ferrari (2009)	0.064	0.064	0.016	128	>32	8	>16	4
Cg27	azole-resistant	blood culture							0.064	0.064	0.016	128	>32	8	>16	4
Cg28	azole-resistant	blood culture	*PDR1*	2626G>T	D876Y	26.0		Sanglard (2016) Ferrari (2009)	0.064	0.064	0.032	128	>32	8	>16	4
			*PDR1*	3236G>T	G1079V	73.04		Ferrari (2009)								
Cg30	azole-resistant	blood culture	*PDR1*	1043G>A	G348D	99.87		Ferrari (2009) Tsai (2010)*	0.064	0.064	0.016	256	>32	8	>16	8
Cg31	azole-resistant	blood culture	*PDR1*	871T>C	L291P	99.95		Ferrari (2009)*	0.064	0.125	0.032	256	>32	8	>16	4
			*PDR1*	872T>C	L291P	99.78		Ferrari (2009)*								
Cg32	azole-resistant	sputum							0.064	0.125	0.064	128	>32	8	>16	4
Cg33	azole-resistant	urine	*PDR1*	1042G>A	G348S	99.8		Ferrari (2009) Tsai (2010)*	0.064	0.064	0.032	256	>32	16	>16	8
Cg34	azole-resistant	not specified	*PDR1*	1114T>C	Y372H	99.83		Ferrari (2009)*	0.064	0.125	0.032	256	>32	8	>16	8
Cg35	azole-resistant	vaginal swab	*PDR1*	1037G>A	G346D	99.74		Ferrari (2009)*	0.064	0.125	0.032	256	>32	8	>16	8
Cg36	azole-resistant	urine	*PDR1*	862C>G	H288D	99.0			0.064	0.125	0.032	256	>32	8	>16	8
Cg37	azole-resistant	ascites	*PDR1*	1037G>C	G346A	99.74		Ferrari (2009)*	0.064	0.125	0.032	256	>32	16	>16	>8
Cg38	azole-resistant	ascites	*PDR1*	1042G>A	G348S	99.72		Ferrari (2009) Tsai (2010) *	0.125	0.064	0.064	256	>32	8	>16	8
Cg39	azole-resistant	feces							0.125	0.064	0.064	256	>32	16	>16	>8
Cg41	echinocandin-resistant	capillary drainage	*FKS2*	1999C>A	P667T	99.7	1 bp after HS1	Spreghini (2012) Garcia-Effron (2009)	0.25	2	0.016	4	0.125	0.032	0.125	0.064
Cg45	echinocandin-resistant	drainage fluid	*FKS2*	1987T>C	S663P	99.0	HS 1	Garnaud (2015) Beyda (2015) Zimbeck (2010)	2	>16	2	4	0.25	0.125	0.25	0.125
Cg51	echinocandin-resistant	blood culture	*FKS2*	1977_1979 delCTT	F659del	99.8	HS 1	Saraya (2014)	2	>16	4	32	2	2	2	0.5
Cg42	borderline echinocandin-resistant	blood culture							0.25	0.125	0.032	8	1	0.25	0.5	0.125
Cg43	borderline echinocandin-resistant	blood culture							0.25	0.125	0.032	8	1	0.25	0.5	0.25
Cg29	multi-resistant	blood culture	*FKS1*	3967A>G	K1323E	99.79	15 bp before HS2		0.125	0.125	0.064	128	16	8	8	8
Cg46	multi-resistant	sterile fluid	*FKS1*	1874T>C	F625S	99.68	HS 1	Garnaud (2015)	1	2	0.25	128	32	8	>16	4
			*PDR1*	889T>C	W297R	99.62		Ferrari (2009)								
Cg47	multi-resistant	feces	*FKS2*	1987T>C	S663P	99.45	HS 1	Garnaud (2015) Beyda (2015) Zimbeck (2010)	4	16	4	128	32	4	>16	4
Cg48	multi-resistant	not specified	*FKS1*	3967A>G	K1323E	99.75	15 bp before HS2		0.25	0.25	0.125	128	8	4	8	4
Cg49	multi-resistant	urine	*PDR1*	985G>T	V329F	99.63		Healey (2016)	0.064	0.125	0.125	128	>32	16	>16	8
Cg50	multi-resistant	not specified	*FKS2*	1976T>C	F659S	98.28	HS1	Garcia-Effron (2009)								
			*PDR1*	3263G>A	G1088E	99.73			0.25	0.5	0.064	256	16	8	4	4

*same position, different aminoacid substitution

### Validation of the sequencing process

For the validation of the sequencing process, different control strains were sequenced and no discrepancies to the published sequences were found. The Q30 quality score was 87% and the total number of reads was 11.7 million. The number of reads of each isolate was between 250,000 and 400,000. The minimal and maximal mean coverages of the genes were 2,250x and 32,000x respectively. NGS data revealed the presence of heterozygous mutations in *C*. *albicans* with a variant (frequency rate) in approximately 50% of the reads (48.2%-51.7%). For homozygous mutations close to 100% (98.6%-100%) of the reads showed the presence of the variant. As *C*. *glabrata* is haploid, variants in the reads of this species had a frequency of approximately 100% (97.9%-100%). In isolate Cg28, we found two mutations with a frequency of 26% and 73%, which could be caused by a subpopulation. This observation was verified with Sanger sequencing. The frequencies of the mutations of all other isolates indicate that they are clonal and without subpopulations.

### Non causal polymorphisms

Mutations present in both susceptible and resistant isolates were defined as polymorphisms non-causal for antifungal resistance development. Of the detected 54 missense mutations, seven were already defined as polymorphisms and four mutations were displayed by susceptible isolates and thus were classified as polymorphisms. The missense mutations S935L [18] and S941P [18] in *TAC1* have already been described as polymorphisms. The heterozygous mutation S937L was also present in two azole-susceptible isolates. This mutation has not been described, and causality in homozygous cases cannot be ruled out. In heterozygous cases, however, this mutation does not cause azole resistance. In *ERG11*, the mutations D116E [18], K128T [26] and E266D [26] were observed homozygous as well as heterozygous in susceptible strains and have already been described as non-causal mutations. The mutation V488I in *ERG11* was described as causal by Manastir et al., and as non-causal by Wang et al. [26, 27]. We found this mutation homozygous in an azole susceptible strain, which supports the study published by Wang et al. In *ERG3* we detected the hitherto unknown homozygous mutations A138T, P181A and the heterozygous P267S, which could also be found in susceptible isolates in our study.

### Potentially causal resistance mutations

In all strains, 87 different silent mutations compared to the reference sequences of the strains CBS138 and SC5314 were found. Fifty different missense mutations as well as an in frame deletion and two premature stop codons were detected. All mutations including silent mutations are listed in the supplementary data (S3 Table). We identified 43 missense mutations as potentially causal, as they were only present in resistant isolates [28]. Of these, 30 have already been described as causative for resistance acquisition. In our study, 13 potentially causal mutations are reported for the first time. Tables 3, 4, 5 and 6 show the potentially causal mutations of the respective genes. Only missense mutations, which are classified as potentially causal, are listed.

**Table 5:** Potentially causal missense mutations in *C*. *albicans* isolates, homozygous mutations in bold.

Gene	Mutation	Literature	susceptible		azole-resistant							echinocandin-resistant						Borderline echinocandin-resistant		multi-resistant	
			Ca1	Ca2	Ca3	Ca5	Ca6	Ca8	Ca9	Ca10	Ca11	Ca13	Ca14	Ca15	Ca16	Ca17	Ca18	Ca19	Ca21	Ca12	Ca22
**TAC1**	**G649D**										**G649D**										
	**N740D**	Siikala et al. 2010						**N740D**													
	**F974L**										**F974L**										
	**N977D**	Coste et al. 2009; Coste et al. 2006			N977D																
	**G980E**	Coste et al. 2009								**G980E**											
**ERG11**	**F72L**	Favre et al. 1999			**F72L**																
	**Y132H**	Favre et al. 1999			**Y132H**			**Y132H**													
	**K143R**	Manastir et al. 2009; Flowers et al. 2015								**K143R**	**K143R**										
	**E208K**								E208K												
	**V437I**	Favre et al. 1999				V437I	**V437I**														
	**G450E**	Favre et al. 1999			**G450E**			**G450E**													
	**T525I**								T525I												
**ERG3**	**A168V**	Morio et al. 2012			**A168V**																
	Y190*																				Y190*
	**S191P**						**S191P**														
	**G261E**								**G261E**												
	Y325*																			Y325*	
	**T329S**	Morio et al. 2012			**T329S**																
	**A353T**	Morio et al. 2012																			**A353T**
**GSC1**	**F641C**											F641C									
	**F641S**	Wiederhold et al. 2011; Balashov et al. 2006											**F641S**								
	**S645P**	Garnaud et al. 2015												**S645P**	S645P	**S645P**					
	**P649H**	Garcia-Effron et al. 2008; Desnos-Ollivier et al. 2008; Dudiuk et al. 2015															**P649H**				
	**M696V**																**M696V**				

**Table 6:** Potentially causal missense mutations in *C*. *glabrata* isolates.

Gene	Mutation	Literature	susceptible		azole-resistant													echinocandin-resistant			Borderline echinocandin-esistant		multi-resistant					
			Cg23	Cg24	Cg26	Cg27	Cg28	Cg30	Cg31	Cg32	Cg33	Cg34	Cg35	Cg36	Cg37	Cg38	Cg39	Cg41	Cg45	Cg51	Cg42	Cg43	Cg46	Cg47	Cg48	Cg49	Cg50	Cg29
**Erg11**	–	–																										
**PDR1**	**H288D**													H288D														
	**L291P**	Ferrari et al. 2009^a^							L291P																			
	**W297R**	Ferrari et al. 2009; Tsai et al 2010																						W297R				
	**V329F**	Healey et al. 2016																									V329F	
	**G346D**	Ferrari et al. 2009^a^											G346D															
	**G346A**	Ferrari et al. 2009^a^													G346A													
	**G348S**	Tsai et al. 2010									G348S					G348S												
	**G348D**	Tsai et al. 2010						G348D																				
	**Y372H**	Ferrari et al. 2009										Y372H																
	**D876Y**	Sanglard und Coste 2016; Ferrari et al. 2009					D876Y																					
	**G1079R**	Ferrari et al. 2009			G1079R																							
	**G1079V**	Ferrari et al. 2009^a^					G1079V																					
	**G1088E**																											G1088E
**FKS1**	**F625S**	Garnaud et al. 2015																						F625S				
	**K1323E**																						K1323E			K1323E		
**FKS2**	**F659del**	Saraya et al. 2014																		F659del								
	**F659S**	Garcia-Effron et al. 2009																									F659S	
	**S663P**	Garnaud et al. 2015; Beyda et al. 2015; Zimbeck et al. 2010																	S663P						S663P			
	**P667T**	Spreghini et al. 2012; Garcia-Effron et al. 2009																P667T										

^a^ same position, different aminoacid substitution

In *C*. *albicans*, all seven azole-resistant strains showed a mutation in the target gene *ERG11*. In *ERG3* and *TAC1* four and five potentially causal mutations could be found respectively. In comparison, no mutations in *ERG11* could be detected in *C*. *glabrata*, but 10 of 13 azole- resistant strains showed a mutation in *CgPDR1*. *GSC1* or *FKS* mutations were found in all echinocandin-resistant isolates. No *FKS* mutations could be found in the four borderline echinocandin-resistant *C*. *albicans* and *C*. *glabrata* isolates. All eight multi-resistant isolates had at least one mutation possibly leading to azole or echinocandin resistance (Table 5).

### Mutations in azole-resistant *C*. *albicans* and *C*. *glabrata*

The seven potentially causal mutations F72L [29], Y132H [29], K143R [27, 30], E208K, V437I [29], G450E [29] and T525I were found in *ERG11* in *C*. *albicans*. Isolate Ca9 showed the two heterozygous mutations E208K and T525I, which have not been described so far. In *ERG3*, we detected the seven potentially causal mutations A168V [14], S191P, G261E, T329S [14] and A353T [14]. The mutation A353T is shown as a homozygous substitution in an azole-resistant strain, and as a heterozygous mutation in the azole-susceptible isolates Ca13 and Ca14. In *TAC1*, the five potentially causal mutations G649D, N740D [31], F974L, N977D [10, 32] and G980E [32] are shown. (Table 5).

In contrast to *C*. *albicans*, none of the azole-resistant *C*. *glabrata* isolates showed any mutations in *ERG11*. In *CgPDR1*, 10 different mutations were observed. H288D has not yet been described. The mutations L291P, G346D, G346A, G348S, G348D and Y372H have been described in these positions but with different amino acid substitutions [33, 34]. The mutations D876Y, G1079R and G1079V have been described as causal by Ferrari et al. [33]. No mutations in the sequenced regions of *CgPDR1* have been detected in the isolates Cg27, Cg32 and Cg39 (Table 6).

### Mutations in echinocandin-resistant *C*. *albicans* and *C*. *glabrata*

In all six echinocandin-resistant *C*. *albicans* isolates, a mutation was found in the target gene *GSC1*. All mutations were detected in *GSC1* HS 1 or its immediate vicinity. The mutations observed were S645P [18], which was detected three times, F641C, F641S [7, 35], P649H [36–38] and M696V. For the isolates Ca13 and Ca16 the mutations F641C and S645P, respectively, are shown as heterozygous. Isolate Ca14 shows the mutation F641S, which was homozygous in this strain and was associated with higher MIC than for isolate Ca13. Isolate Ca18 showed the two homozygous mutations P649H and M696V, which are located in HS 1 and 90 nucleotides downstream of HS 1, respectively. The borderline echinocandin-resistant isolates Ca19 and Ca21, which exhibit a MIC of micafungin of 0.064 mg/l and 0.032 mg/l respectively, did not have any mutations in *GSC1*. Our strains showed no missense mutations in the HS regions of *GSC1* genes whenever the MIC was lower than 0.125 mg/l in anidulafungin and caspofungin, and lower than 0.064 mg/l in micafungin (Table 5).

In the echinocandin-resistant isolates of *C*. *glabrata* we identified the mutations S663P [18, 39, 40], F659del [41–43] in *FKS2*, but none in *FKS1*. The isolate Cg51 showed very high MIC values for anidulafungin (2 mg/l), caspofungin (> 16 mg/l) and micafungin (4 mg/l). In this isolate the deletion F659del in *FKS2* HS 1 was detected. The borderline echinocandin-resistant isolates Cg42 and Cg43 showed no mutations in *FKS1* and *FKS2*. In these isolates the MIC for anidulafungin were twofold dilutions above the breakpoint and for micafungin exactly at the breakpoint (Table 6).

### Mutations in multi-resistant *C*. *albicans* and *C*. *glabrata*

The multi-resistant *C*. *albicans* isolates Ca12 and Ca22 showed the homozygous premature stop codons Y325* and Y190* in *ERG3*, respectively. In both *GSC1* HS regions, no mutations were detected despite elevated echinocandin MIC values (Table 5).

In the six multi-resistant *C*. *glabrata* isolates, five mutations were verified in *FKS* genes. In *FKS1*, the mutations K1323E and F625S [18] could be detected in isolates Cg29 and Cg46, respectively. In *FKS2*, the mutations F659S and S663P could be detected in isolates Cg50 and Cg47 respectively. For three of the six multi-resistant isolates, potentially causal mutations were found in *CgPDR1*: W297R [33, 34] for isolate Cg46, V329F [44] for isolate Cg49 and G1088E for isolate Cg50. Only isolates Cg46 and Cg50 showed a mutation in *FKS* genes and *CgPDR1*. Isolate Cg49, which displayed moderately elevated MIC values for echinocandins showed no mutations in *FKS1 and FKS2* (Table 6).

## Discussion

NGS has previously been shown to successfully detect antifungal resistance mutations in clinically important *Candida* species [18]. With targeted resequencing resistance genes of a high number of isolates can be studied simultaneously. Compared to whole genome sequencing (WGS), this approach reduces sequencing costs, generates more manageable raw data and reduces the burden of data analysis. Investigating 50 isolates the costs would reach 16.000 € using WGS. In this study, the costs were only 3000 €. Thus, the sequencing costs could be reduced to at least 5x due to the targeted resequencing design. Additionally, targeted resequencing results in coverage levels much higher than those achieved with WGS. Thus, this method is very reliable and allows the detection of low frequent variants, e.g. resistant subpopulations. Furthermore, sequencing runs are much faster than with the conventional Sanger method. The sequencing process in our project took about 22 hours for 51 strains with 13 amplicons each. In comparison, using Sanger sequencing on a 4-capillary sequencer, the run time would have been about four weeks. Sanger sequencing is more expensive, and the analysis of this extensive sequencing data would be time-consuming.

In this study, a high number of phenotypically resistant isolates obtained from various centres in Austria and Germany were investigated in order to find mutations causing resistance, and to examine if strains categorized as resistant using the clinical breakpoints possess already described or hitherto unknown resistance mutations. Due to high throughput sequencing, we were able to detect established as well as novel resistance mutations in a large sample of antifungal-resistant strains.

### Azole resistance

Target mutations in *ERG11* were detected only in *C*. *albicans*. In every azole-resistant isolate, potentially causal mutations were detected in this gene. Five of these mutations have already been described as inducing resistance, based on the putative mechanism that the change in the protein sequence leads to a reduced binding affinity of azoles [27, 29, 30]. In contrast to *C*. *albicans*, there were no mutations in *ERG11* in any of the 16 azole-resistant *C*. *glabrata* strains. The absence of *ERG11* mutations in azole-resistant *C*. *glabrata* has already been described [45, 46]. This suggests that our results are in concordance with other investigations and *ERG11* mutations have no impact on azole resistance in our *C*. *glabrata* isolates.

*CgPDR1* appears to be a more important cause for azole resistance in *C*. *glabrata*. *CgPDR1* is a transcription factor, which induces the gene expression of the efflux pumps CgCDR1/2p and CgSNQ2p. In *CgPDR1* 67 gain-of-function mutations have been described hitherto [33]. These mutations were associated with intrinsically high expression of the efflux pumps and specifically were related to azole resistance [12]. In our study, 13 potentially causal mutations were found. In 10 out of 13 azole-resistant strains, at least one mutation was found in *CgPDR1*. Eleven of these mutations are known, the remaining mutations H288D and G1088E have been detected for the first time. Tsai et al. described the domains of *CgPDR1* based on the homology between *S*. *cerevisiae PDR1* and *C*. *glabrata CgPDR1* [34]. The DNA-binding domain is positioned at residues 26–59, the regulatory domain at 322–465 and the activation domain at 903–1107. They found four mutations at residues 280–391. Nine of 13 mutations in *CgPDR1* found in our study were located at residues 288–372, near the putative regulatory domain. Accordingly, these mutations could be associated with altered expression of efflux pumps. Three mutations (G1079R, G1079V and G1088E) were identified in the putative activation domain. Thus, mutations in these regions could be associated with overexpression of drug efflux pumps. To confirm the impact of these mutations, the analysis of the expression levels of efflux pumps could be performed in future studies.

Gain-of-function mutations in the transcription factor *TAC1* lead to inherently increased expression of efflux pumps CDR1p and CDR2p and were associated with azole resistance [47]. In our study, five potentially causal mutations in the sequenced areas of *TAC1* could be identified. Of these, G649D and F974L have not been described so far. However, since none of the sequenced strains showed mutations in *TAC1* only, the relevance of this mechanism could not be clarified in this study.

We could also detect loss-of-function mutations in *ERG3* which presumably leads to azole resistance in combination with moderate echinocandin resistance and is described in the multi resistance section.

### Echinocandin resistance

In our study, mutations in *GSC1*, *FKS1* and *FKS2* were detected in all isolates showing an increase of MIC values (>2 dilution folds above CB), which was associated with complete cross-resistance with the exception of one *C*. *glabrata* isolate. Several missense mutations were found in these isolates. For the isolates with moderately elevated MICs, i.e. one to twofold dilutions above the clinical breakpoint and classified as borderline resistant, neither cross-resistance within the echinocandins nor *FKS* mutations were found.

In our study, a single mutation in the HS regions of the target genes led to complete cross-resistance in the class of echinocandins and was seen for both heterozygous and homozygous mutations. In contrast to this observation, the *C*. *glabrata* isolate Cg41 displayed an isolated susceptibility to micafungin (MIC 0.016 mg/l) whereas anidulafungin showed an elevated MIC of 0.25 mg/l and caspofungin 2 mg/l. In this isolate the already known mutation P667T in *FKS2* HS 1 was detected. The absence of cross-resistance has already been reported [3, 48]. This is of particular interest as anidulafungin has been discussed to serve as a surrogate marker for all echinocandins [49, 50]. In our case, however, micafungin could have been an important therapeutic alternative. These results indicate that changes in conformation of 1,3-β-D-glucan synthase may lead to an incomplete cross-resistance in the class of echinocandins depending on the specific structure of the respective agent. Therefore, susceptibility testing of every echinocandin seems to be preferable to using anidulafungin as an indicator for echinocandin resistance.

The external localization of some regions of the putative transmembrane protein 1,3-β-D-glucan synthase seems to reflect the HS regions [51]. All of our six echinocandin-resistant *C*. *albicans* isolates displayed a mutation in *GSC1* HS 1. No mutations were detected in HS 2. Therefore, HS 1 appears to play a more important role in the development of echinocandin resistance in our *C*. *albicans* strains. The *C*. *albicans* isolate Ca18 showed two homozygous mutations in *GSC1*. P649H is located at the downstream end of HS 1 and M696V is located 90 nucleotides downstream HS 1 and 30 nucleotides upstream HS 3, which is described by Johnson et al. [51]. Thus, it could be assumed that in rare cases other HS regions may be involved in acquisition of echinocandin resistance.

Out of six echinocandin-resistant *C*. *glabrata* strains, four showed a mutation in *FKS2* HS 1. The multi-resistant isolates Cg29 and Cg48 showed the mutation K1323E in *FKS1*, which is five amino acids upstream HS 2. These isolates displayed a minimal rise of MIC values being only onefold dilution above the clinical breakpoint. In these cases, the mutation outside the HS was associated with minimally elevated MIC values.

In isolate Cg51 the in frame deletion F659del in FKS2 HS 1 was detected. This strain showed MIC values in the resistant range for anidulafungin (2 mg/l), caspofungin (> 16 mg/l), and micafungin (4 mg/l). This mutation has already been described associated with very high as well as low MIC values, which might be caused by different expression rates [41–43].

### Multi-resistance

Out of 40 clinical strains, two *C*. *albicans* and six *C*. *glabrata* were multi-resistant. In *C*. *glabrata* strains, only two isolates showed mutations in both *FKS1* and *CgpDR1*, which explains azole and echinocandin resistance. In the other four isolates, only a mutation in either the *FKS* genes or in *CgPDR1* could be detected.

The multi-resistant *C*. *albicans* strains Ca12 and Ca22 only showed a loss-of-function mutation in *ERG3* due to the premature stop codons Y325* and Y190*, which presumably leads to azole resistance and moderate resistance to echinocandins without displaying *FKS* mutations. Although the molecular mechanism for the moderate echinocandin resistance is not clear, this observation is in concordance with Rybak et al. [52]. Thus, the importance of ERG3 loss-of-function mutations for resistance development should not be overlooked.

The fact that a potentially causal mutation for azole and echinocandin resistance is not present in every resistant isolate suggests that different—hitherto unknown—resistance mechanisms are involved.

## Conclusion

NGS proved to be a suitable method to detect resistance mutations. This technique allows a thoroughly, more cost-efficient and much faster sequencing method than conventional Sanger sequencing. Furthermore, the targeted resequencing design enables the investigation of a larger sample size than WGS. In combination with the phenotypic analysis of resistance patterns, conclusions can be drawn about the underlying molecular mechanisms.

We investigated 40 resistant clinical *C*. *albicans* and *C*. *glabrata* isolates and found 30 described and 13 novel mutations in six resistance genes. In addition, a high rate of polymorphisms was found in the coding sequences. This observation underlines the importance of the differentiation between polymorphisms and causal mutations. This applies especially for azole resistance, where several mechanisms can lead to resistance and interact with each other. As a consequence, a SNP database, which includes each variant as well as the phenotype, would be helpful to distinguish between polymorphisms and relevant mutations.

In conclusion, an association between mutations in *FKS* genes and echinocandin resistance can be confirmed. However, the acquisition of azole resistance seems to have multifactorial causes. The mutations in *ERG11* appear to play a role only in *C*. *albicans*. In *C*. *glabrata*, overexpression of efflux pumps is conceivable instead. In *C*. *albicans*, homozygous *ERG3* nonsense mutations seem to be associated with azole resistance and moderately elevated echinocandin MICs. Four of the multi-resistant *C*. *glabrata* isolates showed no underlying mutations for both echinocandin and azole resistance. Hence, there are still other cellular mechanisms, which require further investigations.

## Supporting information

S1 Table *C*. *albicans* primers used in this study. (DOCX)

S2 Table *C*. *glabrata* primers used in this study. (DOCX)

S3 Table List of mutations detected in this study. (DOCX)
