# Supplementary material for: Analysis of antifungal resistance genes in Candida albicans and Candida glabrata using next generation sequencing
Source: PLoS One. 2019 Jan 10;14(1):e0210397. doi: 10.1371/journal.pone.0210397 (PMC6328131; doi:10.1371/journal.pone.0210397)
Supplement: S1 Table — (DOCX) [file pone.0210397.s001.docx]

**S1 Table: C. albicans Primers used in this study**

**Reference:**

Garnaud C, Botterel F, Sertour N, Bougnoux M-E, Dannaoui E, Larrat S, Hennequin C, Guinea J, Cornet M, Maubon D. 2015. Next-generation sequencing offers new insights into the resistance of Candida spp. to echinocandins and azoles. J Antimicrob Chemother 70:2556–2565. doi:10.1093/jac/dkv139.
