# Supplementary material for: Analysis of antifungal resistance genes in Candida albicans and Candida glabrata using next generation sequencing
Source: PLoS One. 2019 Jan 10;14(1):e0210397. doi: 10.1371/journal.pone.0210397 (PMC6328131; doi:10.1371/journal.pone.0210397)
Supplement: S3 Table — (DOCX) [file pone.0210397.s003.docx]

**S3 Table: List of mutations detected in this study**
